# Metabolic dysfunction-associated fatty liver in type 2 diabetes mellitus patient: can a systematic review of and meta-analysis of commonly used TCM-preparation shed light on their efficacy?

**DOI:** 10.3389/fphar.2025.1578371

**Published:** 2025-08-14

**Authors:** Jingsi Cao, Tingting Bao, Jingxian Cao, Xuechun Fan, Yanyan Wang, Jing Yu, Ying Wang, RuiXue Deng, Jingjing Shi, Changlong Shen, Qingwei Li, Jia Mi

**Affiliations:** ^1^ College of Traditional Chinese Medicine, Changchun University of Chinese Medicine, Changchun, China; ^2^ Institute of Metabolic Diseases, Guang’anmen Hospital, China Academy of Chinese Medical Sciences, Beijing, China; ^3^ College of Traditional Chinese Medicine, Ningxia Medical University, Yinchuan, China; ^4^ Department of Endocrinology, The First Affiliated Hospital of Changchun University of Chinese Medicine, Changchun, China; ^5^ Gastroenterology department, Yanji/Yanbian Traditional Chinese Medicine Hospital, Yanji, China

**Keywords:** TCM-preparation, metabolic dysfunction-associated fatty liver disease (MAFLD), type 2 diabetes mellitus (T2DM), meta-analysis, systematic review

## Abstract

**Background:**

Type 2 diabetes mellitus (T2DM) and Metabolic Dysfunction-Associated Fatty Liver Disease (MAFLD) are mutually causal, which can jointly promote the development of the disease. As an important means to evaluate the steatosis of MAFLD, Controlled Attenuation Parameter (CAP) can also evaluate the metabolism related fat parameters. Whether Traditional Chinese Medicine preparation (TCM-preparation) can improve CAP has not been reported yet, so the purpose of this meta-analysis is to evaluate the effect of TCM-preparation on CAP in T2DM patients with MAFLD.

**Methods:**

In this meta-analysis, eight databases were searched from the establishment of the database to 23 April 2025, to obtain clinical randomized controlled trials of TCM-preparation or TCM-preparation combined with Standard Biomedical Treatment in the treatment of T2DM complicated with MAFLD. After screening the literature and extracting the data, Meta-analysis was conducted using RevMan5.4 software, with results presented in the form of a forest plot. This study was registered with PROSPERO (CRD42024569613).

**Results:**

A total of 599 papers were retrieved, and the papers were screened according to the nadir and ranking criteria, and finally 8 trials (648 participants) were included in this study. Meta-analysis showed that TCM-preparation significantly reduced CAP values in patients with T2DM combined with MAFLD compared with TCM-preparation (MD = −15.19, CI [−22.53, −7.85], P < 0.0001).

**Conclusion:**

TCM-preparation can reduce the CAP value of T2DM combined with MAFLD and can have a good therapeutic effect on glucose-lipid metabolism level and liver function, indicating that TCM-preparation can reduce the deposition of hepatic fat, restore the function of hepatocytes, and have a positive effect on T2DM combined with MAFLD; however, due to the heterogeneity of the included literature, it needs to be further validation by high-quality trials.

## 1 Introduction

Metabolic Dysfunction-Associated Fatty Liver Disease (MAFLD) is defined by more than 5% hepatic steatosis, excluding alcohol intake, along with the presence of at least one of the following: overweight/obesity, type 2 diabetes mellitus (T2DM), or metabolic dysregulation (Eslam et al., 2020), which is the main cause of liver disease worldwide (Younossi et al., 2018), affecting 38% of the global population (Wong et al., 2023). At the same time, the incidence of diabetes mellitus (DM) is increasing year by year. About 537 million adults worldwide have DM, of which T2DM accounts for more than 90% (Aguirre et al., 2013). T2DM as MAFLD often occurs at the same time, and can work together to lead to a series of adverse consequences. Studies have shown that up to 75% of T2DM patients have MAFLD, and T2DM can accelerate the progression of MAFLD (Younossi et al., 2018); MAFLD also increases the risk of T2DM and accelerates the progression of complications of T2DM (Stefan and Cusi, 2022). T2DM and MAFLD are mutually causal, which can jointly promote the development of the disease, and the etiology of both are relatively complex, which increases the difficulty of clinical treatment and has a great impact on the quality of life of patients.

Insulin resistance (IR), obesity, and dyslipidemia are associated with the development of T2DM and MAFLD (Leite et al., 2014), both of which are characterized by increased liver fat (Kotronen et al., 2008). Dysfunctions in the uptake, synthesis, oxidation, and secretion of lipids in hepatocytes can lead to hepatic steatosis. Hepatic steatosis is an independent factor in the progression of MAFLD (Castéra et al., 2003; Leandro et al., 2006) and is positively correlated with the onset of T2DM (Tian et al., 2023), potentially influencing the development of diabetes complications (Yanni et al., 2024). Although liver enzymes can reflect liver injury, they are not specific for fatty liver and are easily interfered with by other factors (alcohol, drugs). Currently, ultrasound is recommended as the primary method for diagnosing steatosis, but its accuracy decreases when the degree of hepatic steatosis is less than 20%, and it performs poorly in subjects with a BMI over 40 kg/m^2^ (Eslam et al., 2020). The measurement of the controlled attenuation parameter (CAP) is simple, operator-independent, and cost-effective, with a high sensitivity to steatosis, making it useful for assessing liver steatosis (Thiele et al., 2018). It is considered a standardized, non-invasive method for measuring hepatic steatosis (Karlas et al., 2017) and is significantly associated with metabolic fatty parameters, allowing for the evaluation of metabolic syndrome (de Lédinghen et al., 2014).

Due to the high prevalence and potential risk of T2DM complicated with MAFLD, it has become a top priority to seek positive and effective measures to protect this part of the population, and the treatment of MAFLD is mainly based on lifestyle interventions such as diet and exercise, and there is still a lack of therapeutic drugs for MAFLD. Traditional Chinese medicine (TCM) has a history of thousands of years and has been widely used in the treatment of MAFLD and T2DM. The overall concept of TCM and the idea of syndrome differentiation and treatment make TCM-preparation obtain great advantages in regulating this complex metabolic disease (Dai et al., 2021). TCM’s prevention oriented idea can also prevent the occurrence of diseases and delay the occurrence of complications, which has great advantages for T2DM patients with MAFLD.

TCM-preparation can treat diseases through multiple targets and pathways, which is consistent with the mechanism of action of metabolic diseases. This study (Wang et al., 2022) provides evidence support for TCM-preparation in the treatment of T2DM through an evidence-based map approach. An increasing number of clinical trials have demonstrated the effects of TCM-preparation compounding on weight loss, improvement of disorders of glucose-lipid metabolism and IR (Xu et al., 2022). Studies have shown that TCM-preparation improves glucose transport and utilization, improves glycogen metabolism, promotes GLP-1 release, protects pancreatic islets from damage, and improves intestinal flora, providing a very good therapeutic option for the treatment of T2DM (Ni et al., 2024). With the deeper exploration of TCM-preparation mechanisms, more and more active metabolite mechanisms have been confirmed, for example, quercetin can improve the metabolism of T2DM through multiple pathways such as antioxidant (inhibition of free radicals, enhancement of glutathione and antioxidant enzyme activities), anti-inflammatory (inhibition of NF-κB/COX-2 and other pathways), protection of β-cell function (activation of ERK1/2 to promote insulin secretion), and modulation of glucolipids metabolism (activation of PI3K/PKB, inhibition of adipogenesis) and other multi-pathways to improve diabetes (Li et al., 2022). Ginseng extract can increase the number of new β cells, inhibit inflammatory infiltration in the islets, reduce the proportion of α cells, and increase the proportion of β cells (Yin et al., 2024). Berberine enhances pancreatic islet function in db/db diabetic mice by activating the GLP-1/GLP-1R/PKA signaling pathway in intestinal L cells and pancreatic α cells, thereby promoting the secretion of glucagon-like peptide-1 (GLP-1) (Wu et al., 2023).

At this stage, there is a research systematic evaluation of TCM-preparation on T2DM combined with MAFLD, but it did not report about the effect of CAP value, so this study aims to update the article the latest research by evaluating TCM-preparation on the CAP value and metabolism-related parameters of the patients with T2DM combined with MAFLD, which can provide reference for the future application in the clinic.

## 2 Materials and methods

The design and implementation of this study were carried out with reference to the Cochrane Handbook for systematic reviews. In addition, the review has been registered with Prospero (crd42024569613).

### 2.1 Search strategy

We searched eight databases, including PubMed Embase, the Cochrane Library, Web of Science, CNKI, Wangfang Data, VIP, CBM. And manually search the references that may meet the inclusion criteria to improve the recall rate. The search time is from 23 April 2025.

We used the following words as key words and searched the literature, such as: “type 2 diabetes,” “diabetes,” “non-alcoholic fatty liver disease,” “metabolism related fatty liver disease,” and “traditional Chinese medicine,” Decoction “,” granules “CAP “, “Liver fat attenuation parameters.” The search adopts the combination of free words and subject words, and the corresponding search type is set according to the search characteristics of different databases (Supplementary Table S1).

### 2.2 Inclusion criteria

Study design: This study only included randomized controlled trials (RCTs), and the publication language was limited to Chinese or English;

Participants: T2DM with MAFLD/NAFLD was definitely diagnosed in this study, and there were no restrictions on gender, country, race and age;

Interventions:

Control group: The study received basic treatment or Standard Biomedical Treatment (SBT) treatment (including hypoglycemic drugs, lipid-lowering drugs, liver protection drugs, etc.);

Treatment group: The study received TCM-preparation or TCM-preparation+SBT treatment with unlimited dosage forms (decoction, powder, granule, etc.), and the requirements of TCM-preparation+SBT group were the same as those of the control group.

Outcome measures: The main outcome measure was CAP. The study reported the change results of CAP in the experimental group relative to the control group (or provide sufficient data to calculate these values).

Secondary outcome measures: ① blood glucose related indicators: Fasting Blood Glucose (FPG), Postprandial Blood Glucose (PBG), Glycated hemoglobin A1c (HbA_1c_), Fasting Insulin (FINS), Homeostatic Model Assessment of Insulin Resistance (HOMA-IR); ② Blood lipid related indicators: Triglyceride (TG), Total Cholesterol (TC), Low-Density Lipoprotein Cholesterol (LDL-C), High-Density Lipoprotein Cholesterol (HDL-C); ③ Liver related indicators: Alanine Aminotransferase (ALT), Aspartate Aminotransferase (AST), Liver Stiffness Measurement (LSM); ④ Effective rate and Adverse event rate.

### 2.3 Exclusion criteria

Unable to obtain full text or incomplete data;

Receive other TCM-preparation treatment, including acupuncture, massage, etc.

### 2.4 Study selection and data extraction

According to the search strategy, the retrieved literature was managed using endnote software. Two researchers (JSC and JXC) read the titles and abstracts, screened the literature for the first time. According to the acceptance and exclusion criteria, they screened the literature for the second time by reading the full text; In case of disagreement, consult with the third researcher (QWL).

Two researchers (TTB and XCF) jointly formulated the extraction form and separately extracted the following data, including the first author, publication year, sample size, age, sex, disease duration, intervention measures, medication duration, adverse events and outcome indicators of the experimental group and the control group. After data extraction, the two researchers will review. If there is any inconsistency, they will discuss with the third researcher (JM) and solve it.

### 2.5 Quality assessment

Two researchers (JSC and YW) referred to the Cochrane risk bias assessment manual to evaluate the quality of the included literature. The evaluation includes the following seven items: (1) Random sequence; (2) Allocation concealment; (3) Investigator blinded; (4) The outcome assessors blinded; (5) Completeness of outcome data; (6) Selective reporting; (7) There were other biases. For the above seven items, three kinds of evaluation were given: low risk of bias, high risk of bias and unknown risk of bias. In case of disagreement, it was resolved through consultation with the third investigator (TTB), and the corresponding offset risk map was drawn using Revman5.4 software.

### 2.6 Statistical analysis

Revman5.4 software was used to evaluate the effect of TCM-preparation on T2DM with MAFLD from the aspects of CAP, blood glucose, blood lipids, liver function and etc. Relative risk (RR) was used as effect value for dichotomous variables; Continuous variables were expressed by mean difference (MD) or standardized mean difference (SMD) and their 95% confidence interval (CI). The heterogeneity among studies was evaluated using X^2^ and I^2^. If the heterogeneity among studies was small (P > 0.10, I^2^ < 50%), the fixed effect model was used for combined analysis; On the contrary, the random effects model is used for the combined analysis, and the results are shown in the forest map.

## 3 Result

### 3.1 Search results

**FIGURE 1:**
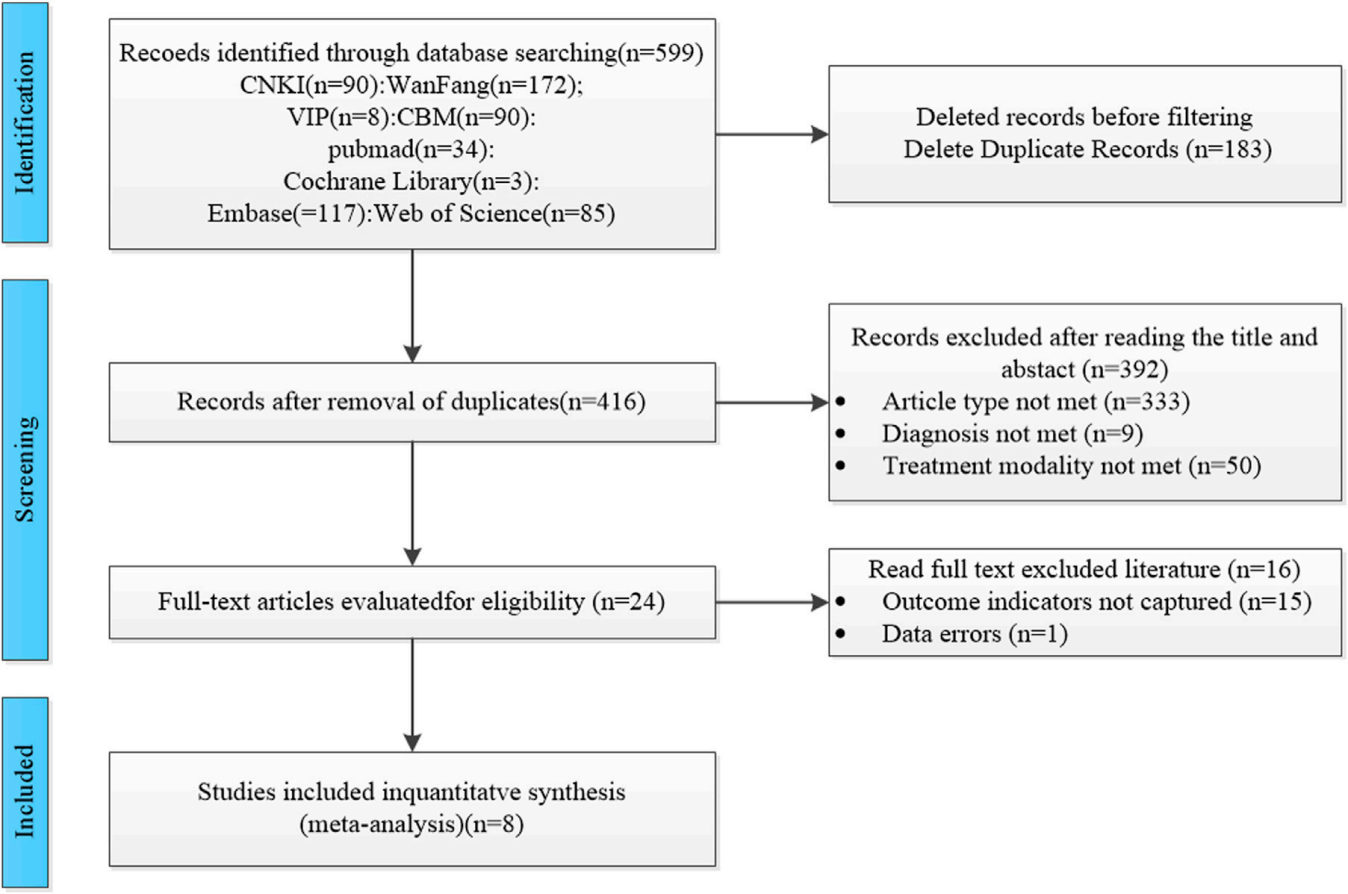
Literature screening flowchart.

A total of 599 studies were included in this study, and we excluded 183 duplicate studies. Among the remaining 416 studies, 333 studies were excluded due to the type of study not being available, including the exclusion of reviews, animal experiments, and summaries of experience, 9 studies were excluded due to the type of treatment not being available, and 50 studies were excluded due to the type of treatment not being available. We excluded 16 papers after carefully reading the full text, 15 papers without obtaining the main outcome indicators, and 1 paper with unreasonable methods of testing. A total of 8 studies (Cai et al., 2023; Cai et al., 2022; Li et al., 2022; Qlan et al., 2023; Wang Y. et al., 2024; Wang S. et al., 2024; Wu et al., 2025; Zhu, 2023) were finally included, and the screening process is shown in Figure 1.

### 3.2 Study characteristics

A total of 8 studies were included, only 1 study was published in English, and the remaining 7 studies were published in Chinese, involving a total of 648 patients, of which 324 were in the treatment group and 324 in the control group, and all the trials were RCTs conducted in China from 2022 to 2025. In these studies, 2 of the treatment groups examined the therapeutic measure as TCM-preparation, and 6 of the studies examined the therapeutic measure as TCM-preparation+SBT, which is the a combination of Chinese and Western medicine; 3 of the TCM-preparation groups took TCM-preparation orally as granules, and 5 of the TCM-preparation groups took TCM-preparation orally as soup. The control group had 3 interventions, 4 studies used hypoglycemic agents, and 2 studies used hypoglycemic agents in combination with lipid-lowering agents. Two studies depended on the patient’s condition; the duration of the interventions was 2–3 months. The specific information of the 8 studies is shown in Table 1. We referred to (http://mpns.kew.org/mpns-portal/ or http://www.plantsoftheworldonline.org) to write down detailed information on all TCM-preparation ingredients. The relevant results are shown in Table 2. Specific information on all Chinese herbal medicines (including family and genus) is shown in Supplementary Table S2.

**TABLE 1:** Characteristics of the included studies.

Study	Sample size (T/C)	Age(Y)		Gender (F/M)		Intervention		Duration	Outcome
		T	C	T	C	T	C		
(Cai et al., 2022)	40/40	51.10 ± 7.74	51.47 ± 9.55	24/16	26/14	TCM-preparation granules+liraglutide (0.6–1.8 mg, qd)	Liraglutide (0.6–1.8 mg, qd)	12 w	①②④⑤⑥⑦⑧⑨⑩⑪⑫⑬⑭⑮
(Cai et al., 2023)	66/66	47.45 ± 10.48	48.09 ± 10.37	36/30	35/31	TCM-preparation granules+melbine (0.5 g, qid)+Atorvastatin calcium (10 mg, qd)	Melbine (0.5 g, qid)+Atorvastatin calcium (10 mg, qd)	12 w	①②⑤⑥⑦⑧⑪⑫⑬⑮⑯
(Zhu, 2023)	48/48	56.91 ± 2.37	57.15 ± 2.46	22/26	25/23	TCM-preparation decoction+melbine (0.5 g, qid)+Atorvastatin calcium (20 mg, qd)	Melbine (0.5 g, qid)+Atorvastatin calcium (20 mg, qd)	8 w	①②④⑥⑨⑫⑬⑯
(Wang S. et al., 2024)	35/35	57.89 ± 14.34	63.23 ± 9.26	19/16	17/18	TCM-preparation decoction+Depending on the condition	Depending on the condition	12 w	①②③④⑩⑭⑮⑯
(Wu et al., 2025)	20/20	56.05 ± 9.47	61 ± 8.39	8/12	45911	TCM-preparation decoction+melbine (0.5 g, qid)	melbine (0.5 g, qid)	12 w	①②③④⑤⑥⑦⑧⑨⑩⑪⑫⑭⑮⑯
(Qian et al., 2023)	47/47	59.19 ± 15.38	51.24 ± 15.15	27/20	25/22	TCM-preparation decoction+melbine (0.5 g, qid)	melbine (0.5 g, qid)	8 w	①②③④⑤⑥⑦⑧⑨⑩⑪⑫⑯
(Li et al., 2022)	38/38	55.96 ± 3.87	56.13 ± 4.05	25/13	22/16	TCM-preparation decoction+melbine (0.5 g, qid)+Atorvastatin calcium (10 mg, qd)+Depending on the condition	melbine (0.5 g,qid)+Atorvastatin calcium (10 mg, qd)+Depending on the condition	12 w	①②④⑪⑫⑬⑮⑯
(Wang Y. et al., 2024)	30/30	41.9 ± 9.3	42.3 ± 8.7	17/13	16/14	TCM-preparation granules	melbine (0.5 g, qid)	12w	①②③④⑥⑦⑧⑨⑩⑪⑫⑭⑮

Abbreviations: T:treatment; C:control; F:female; M:male; W:week; ① CAP; ② FBG; ③ PBG; ④ HbA_1c_; ⑤ FINS; ⑥ HOMA-IR; ⑦ TC; ⑧ TG; ⑨ LDL-C; ⑩ HDL-C; ⑪ ALT; ⑫ AST; ⑬ LSM; ⑭ BMI; ⑮ Effective rate; ⑯ Adverse Event rate.

**TABLE 2:** Characteristics of included interventions.

Study	TCM-preparation formula (dosage, frequency)	Ingredients of TCM-preparation formula	Adverse event
(Cai et al., 2022)	Jinlida granules (Shijiazhuang Ealing Pharmaceutical Co., Ltd., 9 g, tid)	Panax ginseng C.A.Mey, Polygonatum sibiricum Redouté, Atractylodes lancea (Thunb.) DC, Sophora albescens (Rehder) C.Y.Ma, Ophiopogon japonicus (Thunb.) Ker Gawl, Rehmannia glutinosa (Gaertn.) Libosch. ex DC, Reynoutria multiflora (Thunb.) Moldenke, Cornus officinalis Siebold & Zucc, Wolfiporia cocos (Schwein.) Ryvarden & Gilb, Eupatorium japonicum Thunb, Coptis chinensis Franch, Anemarrhena asphodeloides Bunge, Epimedium brevicornu Maxim, Salvia miltiorrhiza Bunge, Pueraria montana var. thomsonii (Benth.) M.R.Almeida, Litchi chinensis Sonn, Lycium chinense Mill	Unreported
(Cai et al., 2023)	Jinlida granules (Shijiazhuang Yiling Pharmaceutical Co. Ltd., National Medicine permission no. Z20050845, 9 g, tid)	Panax ginseng C.A.Mey, Polygonatum sibiricum Redouté, Atractylodes lancea (Thunb.) DC, Sophora albescens (Rehder) C.Y.Ma, Ophiopogon japonicus (Thunb.) Ker Gawl, Rehmannia glutinosa (Gaertn.) Libosch. ex DC, Reynoutria multiflora (Thunb.) Moldenke, Cornus officinalis Siebold & Zucc, Wolfiporia cocos (Schwein.) Ryvarden & Gilb, Eupatorium japonicum Thunb, Coptis chinensis Franch, Anemarrhena asphodeloides Bunge, Epimedium brevicornu Maxim, Salvia miltiorrhiza Bunge, Pueraria montana var. thomsonii (Benth.) M.R.Almeida, Litchi chinensis Sonn, Lycium chinense Mill	1 dizziness
(Zhu, 2023)	Shenling Baizhu Powder is flavored with Erchen decoction (Hospitals are uniformly prepared as a concentrate, 150 mL, tid)	Atractylodes macrocephala Koidz, Wolfiporia cocos (Schwein.) Ryvarden & Gilb, Codonopsis pilosula (Franch.) Nannf, Massa Medicata Fermentata, Dolomiaea costus (Falc.) Kasana & A.K.Pandey, Dioscorea oppositifolia, Lablab purpureus subsp. Purpureus, Glycyrrhiza uralensis Fisch. ex DC, Zingiber officinale Roscoe, Cinnamomum cassia Presl, Pinellia ternata (Thunb.) Makino, Paeonia lactiflora Pall, Citrus reticulata Blanco, Cyperus rotundus L, Perilla frutescens (L.) Britton, Coix lacryma-jobi var. ma-yuen (Rom.Caill.) Stapf, Astragalus mongholicus Bunge, Wurfbainia villosa var. xanthioides (Wall. ex Baker) Škorničk. & A.D.Poulsen	2 gastrointestinal reactions, 1 hypoglycemia, 3 malaise, 1 headache and dizziness
(Wang S. et al., 2024)	Yixiao formula (decoction of water and juice, 1 dose per day, bid)	Astragalus mongholicus Bunge, Pseudostellaria heterophylla (Miq.) Pax, Ophiopogon japonicus (Thunb.) Ker Gawl, Schisandra chinensis (Turcz.) Baill, Salvia miltiorrhiza Bunge, Angelica sinensis (Oliv.) Diels, Paeonia lactiflora Pall, Euonymus alatus (Thunb.) Siebold, Fritillaria thunbergii Miq, Prunella vulgaris L.	No adverse reactions
(Wu et al., 2025)	Dispelling fat and lowering sugar formula (decoction of water to extract juice, 1 dose per day, 150 mL tid)	Astragalus mongholicus Bunge, Bupleurum chinense DC, Citrus × aurantium f. aurantium, Paeonia lactiflora Pall, Crataegus pinnatifida var. pinnatifida, Panax notoginseng (Burkill) F.H.Chen, Salvia miltiorrhiza Bunge, Wolfiporia cocos (Schwein.) Ryvarden & Gilb, Atractylodes macrocephala Koidz, Dendrobium nobile Lindl, Curcuma longa L, Coptis chinensis Franch, Citrus reticulata Blanco	No adverse reactions
(Qian et al., 2023)	Huanglian wendan decoction (Hospitals are uniformly prepared as a concentrate, 150 mL, tid)	Coptis chinensis Franch, Scutellaria baicalensis Georgi, Pinellia ternata (Thunb.) Makino, Bambusa tuldoides Munro, Curcuma phaeocaulis Valeton, Citrus reticulata Blanco, Fritillaria thunbergii Miq, Wolfiporia cocos (Schwein.) Ryvarden & Gilb, Atractylodes macrocephala Koidz, Bupleurum chinense DC, richosanthes kirilowii Maxim, Pueraria montana var. thomsonii (Benth.) M.R.Almeida, Salvia miltiorrhiza Bunge	No adverse reactions
(Li et al., 2022)	Shenmai Lanling Decoction (decoction of water to extract juice, 1 dose per day, 150 mL tid)	Phytolacca americana L, Wolfiporia cocos (Schwein.) Ryvarden & Gilb, Anemarrhena asphodeloides Bunge, Schisandra chinensis (Turcz.) Baill, Pseudostellaria heterophylla (Miq.) Pax, Pinellia ternata (Thunb.) Makino, Lycopus europaeus L, Dioscorea oppositifolia L, Ophiopogon japonicus (Thunb.) Ker Gawl	3 nausea and vomiting, 1 dizziness, 1 hypoglycemia
(Wang Y. et al., 2024)	Wuling powder (Beijing Kang Rentang Pharmaceutical Co., Ltd. formulated granules, 1 bag, bid)	Alisma plantago-aquatica subsp. orientale (Sam.) Sam, Wolfiporia cocos (Schwein.) Ryvarden & Gilb, Polyporus umbellatus (Pers.) Fr, Atractylodes macrocephala Koidz, Cinnamomum cassia Presl	Unreported

### 3.3 Quality assessment

**FIGURE 2:**
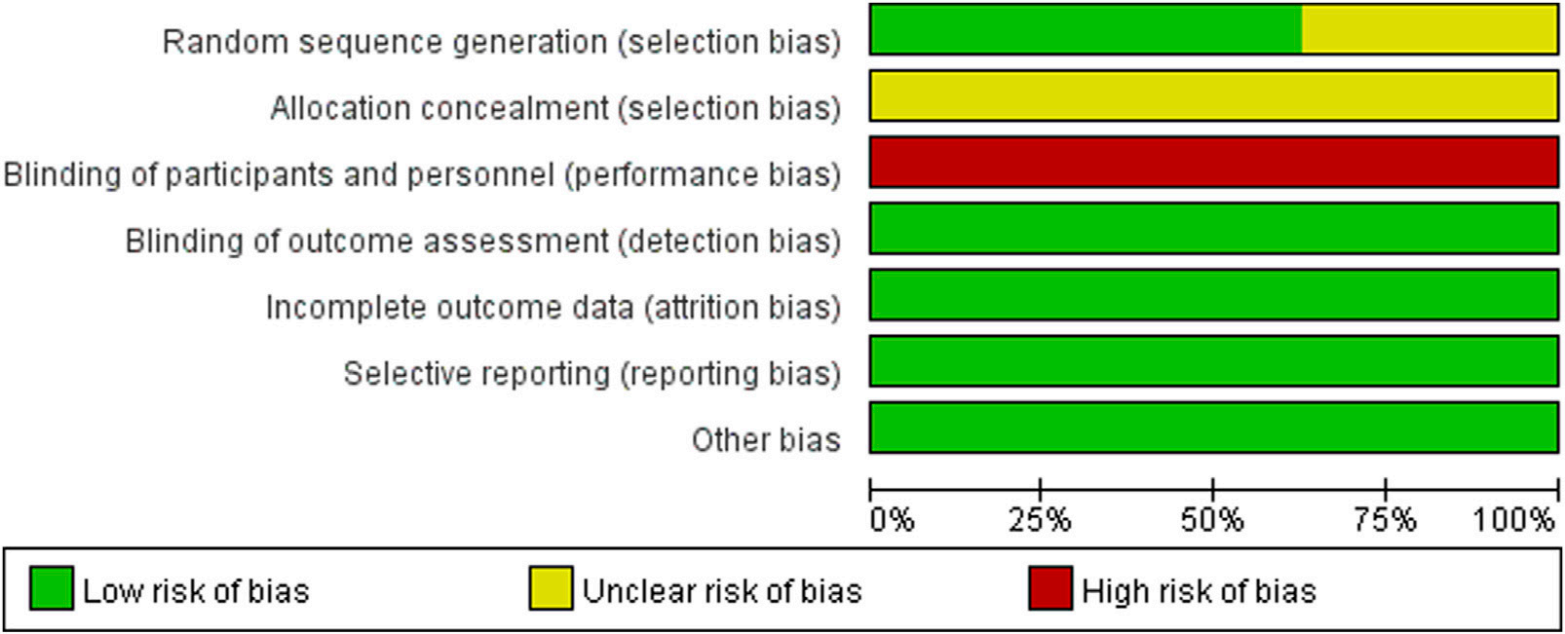
Risk of bias graph.

**FIGURE 3:**
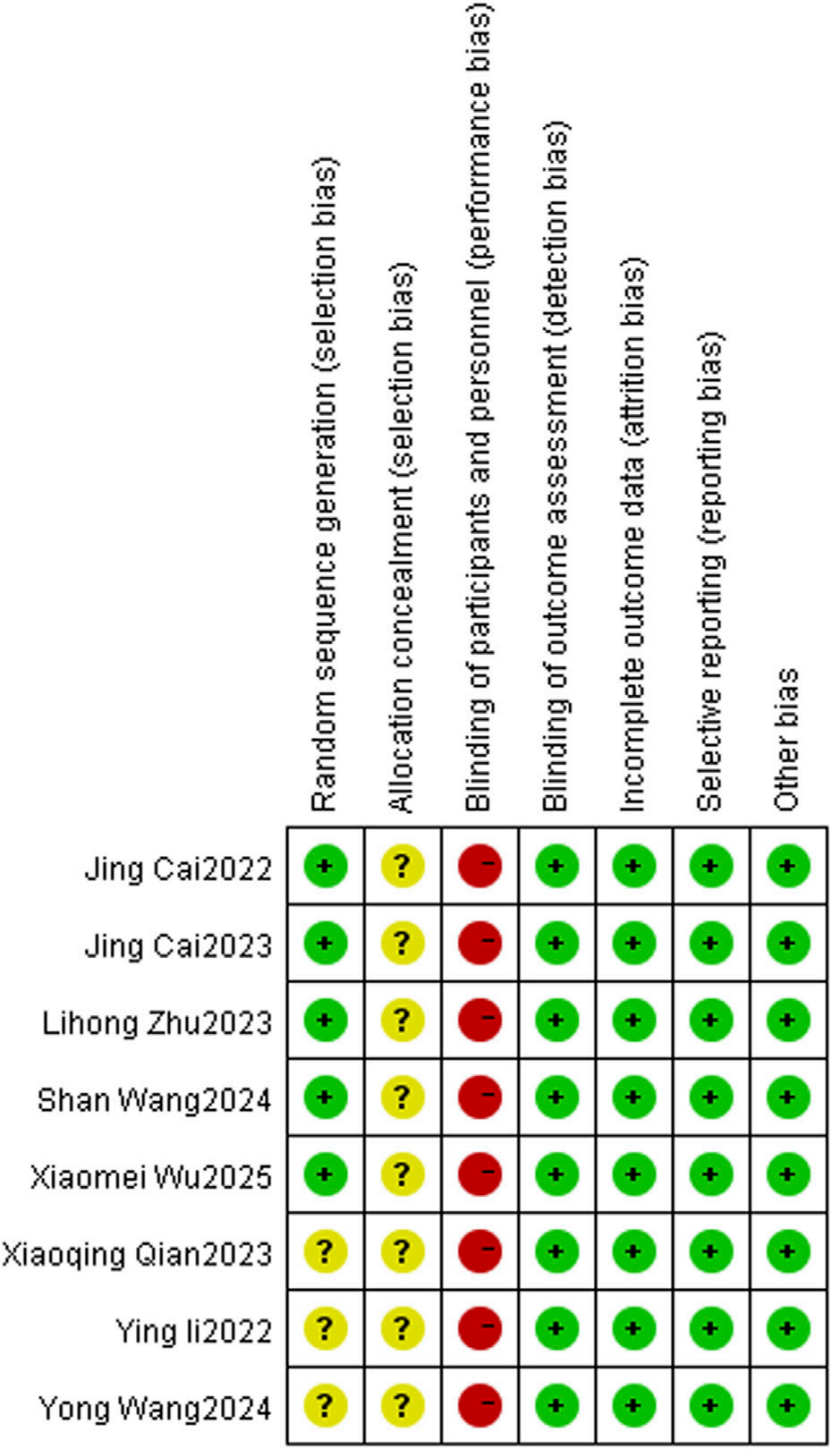
Risk of bias summary.

Of the 8 studies included in the literature, 4 studies used a random number table method and 1 study used a randomized bicolour method, and these 5 studies were categorized as being at low risk of bias; 3 studies reported randomization only, without specifying the details, and were categorized as being at unknown risk of bias. All studies lacked a description of allocation concealment, so the program was rated as “unknown risk of bias”; none of the studies reported blinding, and blinding of investigators and subjects was rated as “high risk of bias,” but all metrics were measured using specific instruments, so metric assessment may not be affected by the risk of bias. However, all indicators were measured using specific instruments, and therefore the assessment of the indicators may not have been affected by blinding, so blinding of the outcome indicators was rated as “low risk of bias”; The studies all reported complete data, incomplete outcome data and Selective reporting were rated as “low risk of bias”; the baseline level of data in this study was homogeneous, so other risks of bias were rated as “Low risk of bias.” About the results of the quality risk assessment, as shown in (Figure 2 and Figure 3).

### 3.4 Meta analysis results

#### 3.4.1 Effect of TCM-preparation on CAP

**FIGURE 4:**
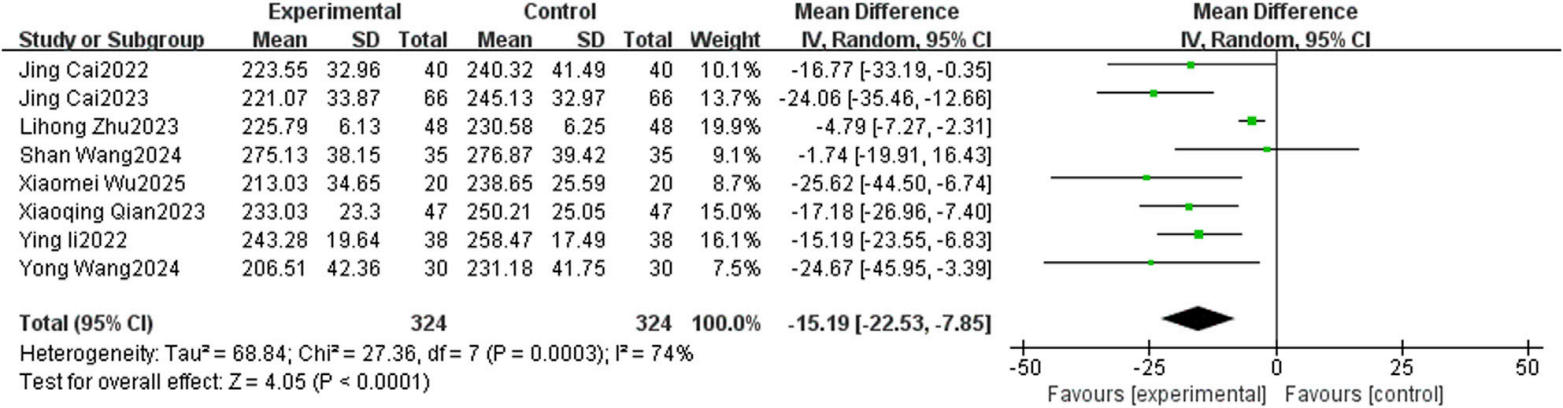
The forest plot (effect size and 95% confidence interval) summarizes the effect of TCM-preparation on CAP.

A total of 8 studies reported the level of CAP, with a large heterogeneity among studies (I^2^ = 74%, P = 0.0003), and Meta-analysis was performed using a random-effects model. The overall study showed that TCM-preparation reduced CAP values in patients with MAFLD combined with T2DM (MD = −15.19, CI [−22.53, −7.85], P < 0.0001) (Figure 4). To explore the source of heterogeneity, we performed subgroup analyses of the intervention duration (8 w, 12 w) and the intervention in the treatment group (TCM-preparation, TCM-preparation+SBT), which suggested that the above reasons might not be the source of heterogeneity (Supplementary Figure S1). Due to the limited number of articles, we did not perform meta-regression. In order to assess the robustness of the results, we excluded articles one by one and analyzed the remaining articles by sensitivity analysis, and found that the heterogeneity was significantly reduced after excluding Lihong Zhu 2023 and the combined effect sizes did not change significantly, suggesting that the results were robust (Table 3). Despite the high heterogeneity, our results still suggest that TCM-preparation can reduce the CAP of T2DM combined with MAFLD.

**TABLE 3:** Exclusion analysis of outcome measures with high heterogeneity.

		Before study exclusion		After study exclusion	
Outcome indicator	Excluded study	Effects model	MD (95% CI)	Effects model	MD (95% CI)
CAP	Zhu, 2023	Random-effects model	−15.19 [−22.53, −7.85]	Fixed-effects model	−17.55 [−22.31, −12.79]
FBG	Wang S. et al., 2024 Li et al., 2022	Random-effects model	−0.68 [−0.97, −0.39]	Fixed-effects model	−0.42 [−0.56, −0.27]
HbA_1c_	Li et al., 2022	Random-effects model	−0.51 [−0.81, −0.22]	Fixed-effects model	−0.42 [−0.54, −0.31]
HOMA-IR	Zhu, 2023	Random-effects model	−0.57 [−0.86, −0.28]	Fixed-effects model	−0.66 [−0.76, −0.56]
LDL	Zhu, 2023	Random-effects model	−0.30 [−0.50, −0.10]	Fixed-effects model	−0.40 [−0.54, −0.26]
HDL	(Wu et al., 2025)	Random-effects model	0.04 [−0.09, 0.17]	Fixed-effects model	0.10 [0.02, 0.18]
AST	Zhu, 2023	Random-effects model	−4.09 [−5.82, −2.36]	Fixed-effects model	−5.05 [−5.95, −4.16]
LSM	Zhu, 2023	Random-effects model	−1.20 [−1.96, −0.45]	Fixed-effects model	−1.55 [−2.04, −1.05]
BMI	Wang S. et al., 2024 Wang Y. et al., 2024	Random-effects model	−1.16 [−2.32, 0.00]	Fixed-effects model	−1.10 [−1.79, −0.42]

#### 3.4.2 Effect of TCM-preparation on blood glucose control

##### 3.4.2.1 FPG

**FIGURE 5:**
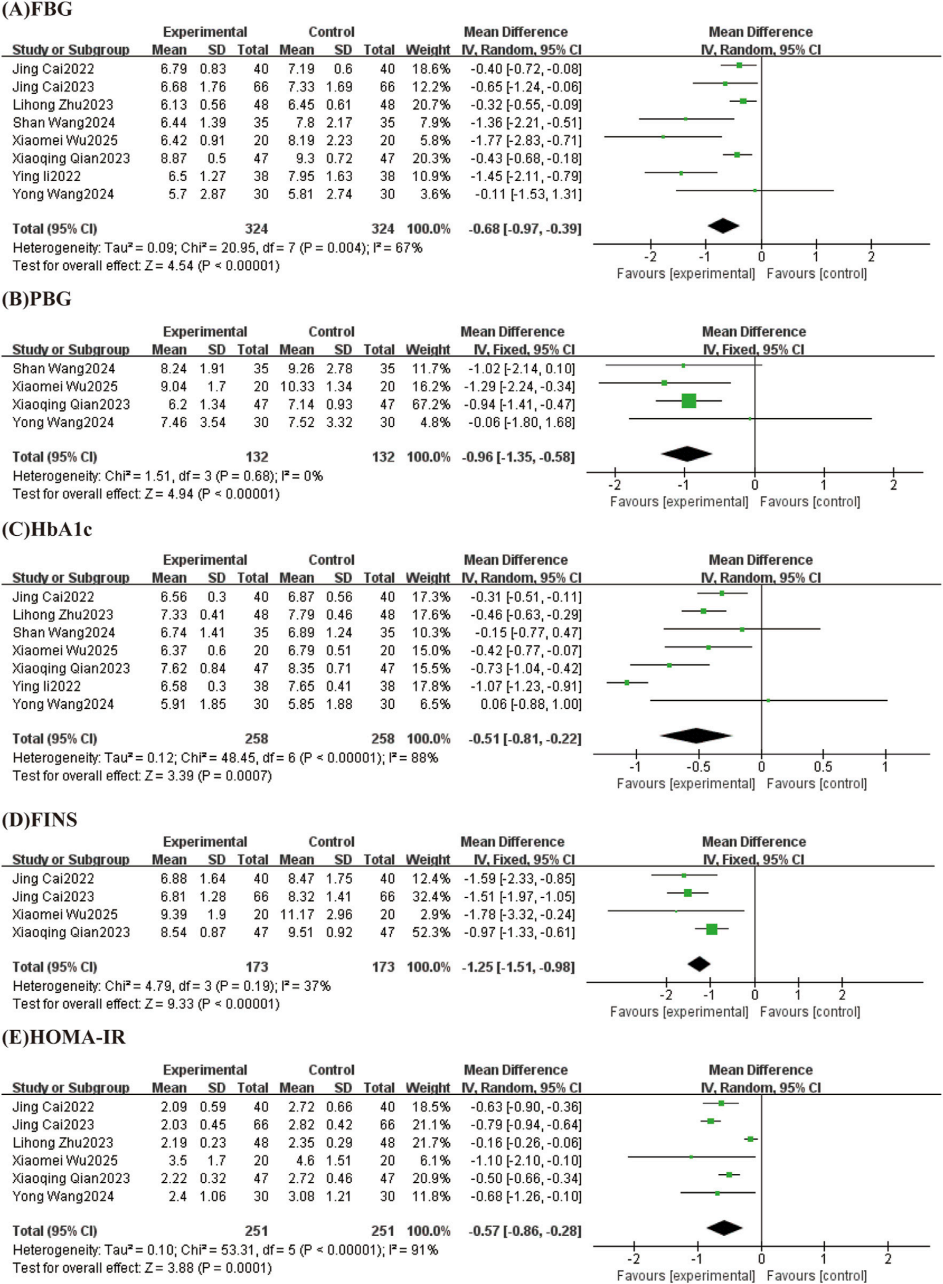
Forest plots (effect size and 95% confidence interval) summarize the effects of TCM-preparation on five outcome measures: **(A)** FPG, **(B)** PBG, **(C)** HbA1c, **(D)** FINS, **(E)** HOMA-IR.

Eight studies reported levels of FBG, with a high degree of heterogeneity between studies (I^2^ = 67%, p = 0.004), which were analyzed by Meta-analysis using a random-effects model. The overall study showed that TCM-preparation reduced FBG in patients with T2DM combined with MAFLD (MD = −0.68, CI [−0.97, −0.39], P < 0.00001) (Figure 5A). To explore the source of heterogeneity, we performed subgroup analyses of intervention duration (8 w, 12 w) and intervention in the treatment group (TCM-preparation, TCM-preparation+SBT), which suggested that the above reasons might not be the source of heterogeneity (Supplementary Figure S2). In order to assess the robustness of the results, we excluded articles one by one and analyzed the remaining articles by sensitivity analysis, and found that the heterogeneity was significantly reduced after excluding Shan Wang 2024, Ying Li 2022, and the combined effect sizes did not change significantly, suggesting that the results were of a robust type (Table 3).

##### 3.4.2.2 PBG

Results from 4 studies reported PBG with low heterogeneity between studies (I^2^ = 0%, P = 0.68) using a fixed effect model. The results showed that TCM-preparation reduced PBG in patients with T2DM combined with MAFLD (MD = −0.96, CI [−1.35, −0.58], P < 0.00001) (Figure 5B).

##### 3.4.2.3 HbA_1c_

Seven studies reported levels of HbA_1c_, with large heterogeneity between studies (I^2^ = 88%, P < 0.00001), and Meta-analysis was performed using a random effects model. The overall study showed that TCM-preparation reduced HbA_1c_ in patients with T2DM combined with MAFLD (MD = −0.51, CI [−0.81, −0.22], P = 0.0007) (Figure 5C). To explore the source of heterogeneity, we performed subgroup analyses of intervention duration (8 w, 12 w) and treatment group interventions (TCM-preparation, TCM-preparation+SBT), which suggested that the above reasons might not be the source of heterogeneity (Supplementary Figure S3). To assess the robustness of the results, we excluded articles one by one and analyzed the remaining articles by sensitivity analysis, and found that heterogeneity was significantly reduced after excluding Ying Li 2022 and the combined effect sizes did not change significantly, suggesting that the results were robust (Table 3).

##### 3.4.2.4 FINS

FINS was reported in 4 studies with low heterogeneity between studies (I^2^ = 37%, P = 0.19) using a fixed effect model. The results showed that TCM-preparation improved FINS in patients with T2DM combined with MAFLD (MD = −1.25, CI [−1.51, −0.98], P < 0.00001) (Figure 5D).

##### 3.4.2.5 HOMA-IR

Six studies reported the level of HOMA-IR, with a high degree of heterogeneity between studies (I^2^ = 91%, P < 0.00001), and Meta-analysis was performed using a random-effects model. The overall study showed that TCM-preparation reduced HOMA-IR in patients with T2DM combined with MAFLD (MD = −0.57, CI [−0.86, −0.28], P = 0.0001) (Figure 5E). To explore the source of heterogeneity, we performed subgroup analyses of intervention time (8 w, 12 w), which suggested that intervention time may not be a source of heterogeneity (Supplementary Figure S4). To assess the robustness of the results, we excluded articles one at a time and analyzed the remaining articles by sensitivity analysis, and found that heterogeneity was significantly reduced after excluding Lihong Zhu 2023, and the combined effect sizes did not change significantly, suggesting that the results were robust (Table 3).

#### 3.4.3 Effect of TCM-preparation on lipid index

##### 3.4.3.1 TC

**FIGURE 6:**
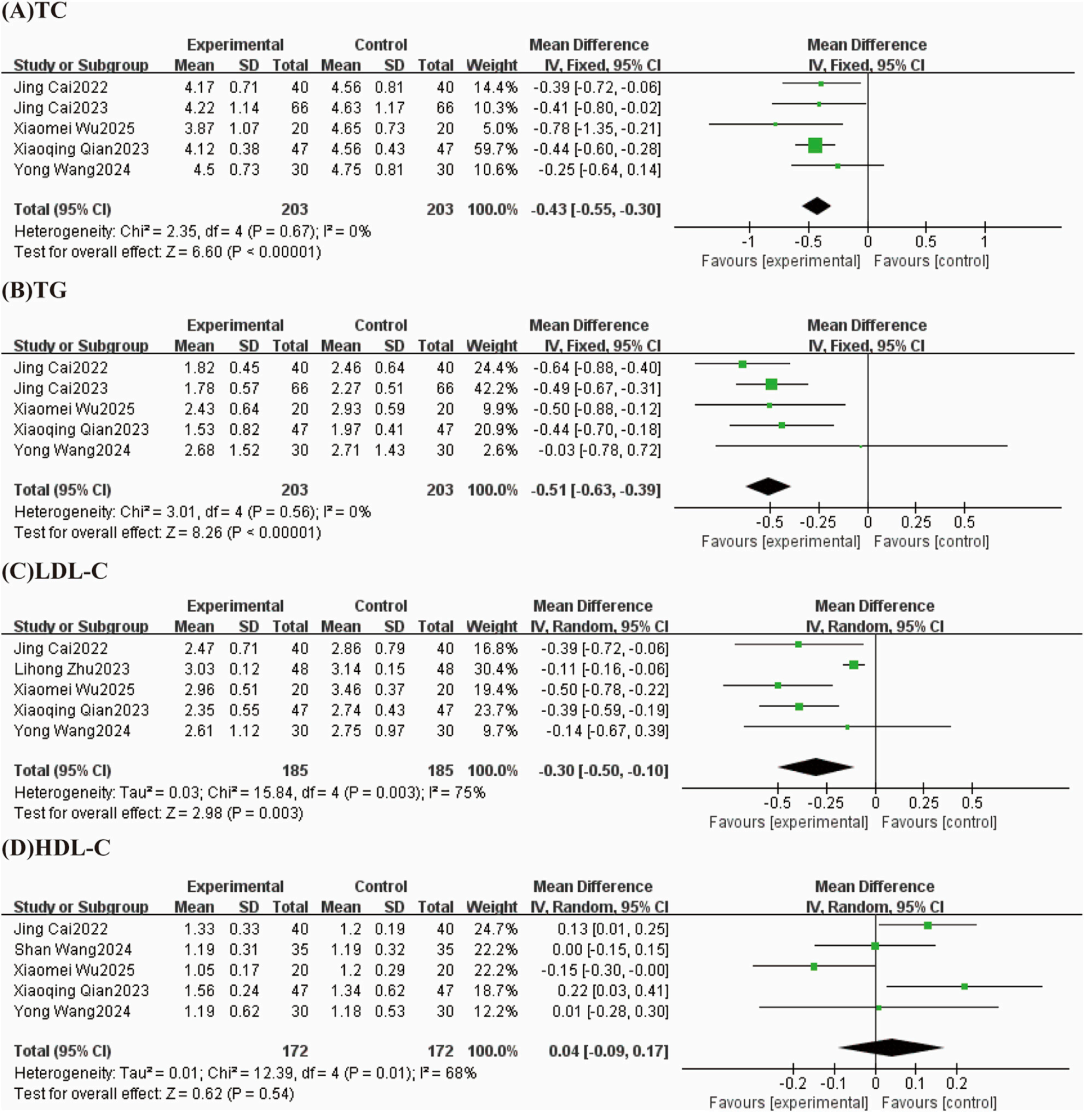
Forest plots (effect size and 95% confidence interval) summarize the effects of TCM-preparation on four outcome measures: **(A)** TC, **(B)** TG, **(C)** LDL-C, **(D)** HDL-C.

Five studies reported TC with low heterogeneity between studies (I^2^ = 0%, P = 0.67) using a fixed effect model. The results showed that TCM-preparation improved TC in patients with T2DM combined with MAFLD (MD = −0.43, CI [−0.55, −0.30] P < 0.00001) (Figure 6A).

##### 3.4.3.2 TG

The results of 5 studies reported TG with low heterogeneity between studies (I^2^ = 0%, P = 0.56) using a fixed effect model. The results showed that TCM-preparation improved TG in patients with T2DM combined with MAFLD (MD = −0.51, CI [−0.63, −0.39] P < 0.00001) (Figure 6B).

##### 3.4.3.3 LDL-C

LDL-C were reported in 5 studies, with a high degree of heterogeneity between studies (I^2^ = 75%, p = 0.003), and Meta-analysis was performed using a random-effects model. The overall study showed that TCM-preparation reduced LDL-C in patients with T2DM combined with MAFLD (MD = −0.30, CI [−0.50, −0.10], P = 0.003) (Figure 6C). To explore the source of heterogeneity, we performed subgroup analyses of intervention time (8 w, 12 w), which suggested that intervention time may not be a source of heterogeneity (Supplementary Figure S5). To assess the robustness of the results, we excluded articles one at a time and analyzed the remaining articles by sensitivity analysis, and found that heterogeneity was significantly reduced after excluding Lihong Zhu 2023 and the combined effect size did not change significantly, suggesting that the results were robust (Table 3).

##### 3.4.3.4 HDL-C

Five studies reported levels of HDL-C, with a high degree of heterogeneity between studies (I^2^ = 68%, p = 0.01), which were analyzed by Meta-analysis using a random-effects model. The overall study showed that TCM-preparation had the same effect of improving HDL-C in patients with T2DM combined with MAFLD compared with controls (MD = 0.04, CI [−0.09, 0.17], P = 0.54) (Figure 6D). To explore the source of heterogeneity, we performed subgroup analyses of the interventions (TCM-preparation, TCM-preparation+SBT) in the treatment group, which suggested that the treatment group interventions may not be the source of heterogeneity (Supplementary Figure S6). To assess the robustness of the results, we excluded articles one at a time and analyzed the remaining articles by sensitivity analysis, and found that heterogeneity was significantly reduced by excluding Xiaomei Wu 2025, but the amount of the combined effect changed significantly, suggesting that the results were relatively unstable (Table 3).

#### 3.4.4 Effect of TCM-preparation on liver function

##### 3.4.4.1 ALT

**FIGURE 7:**
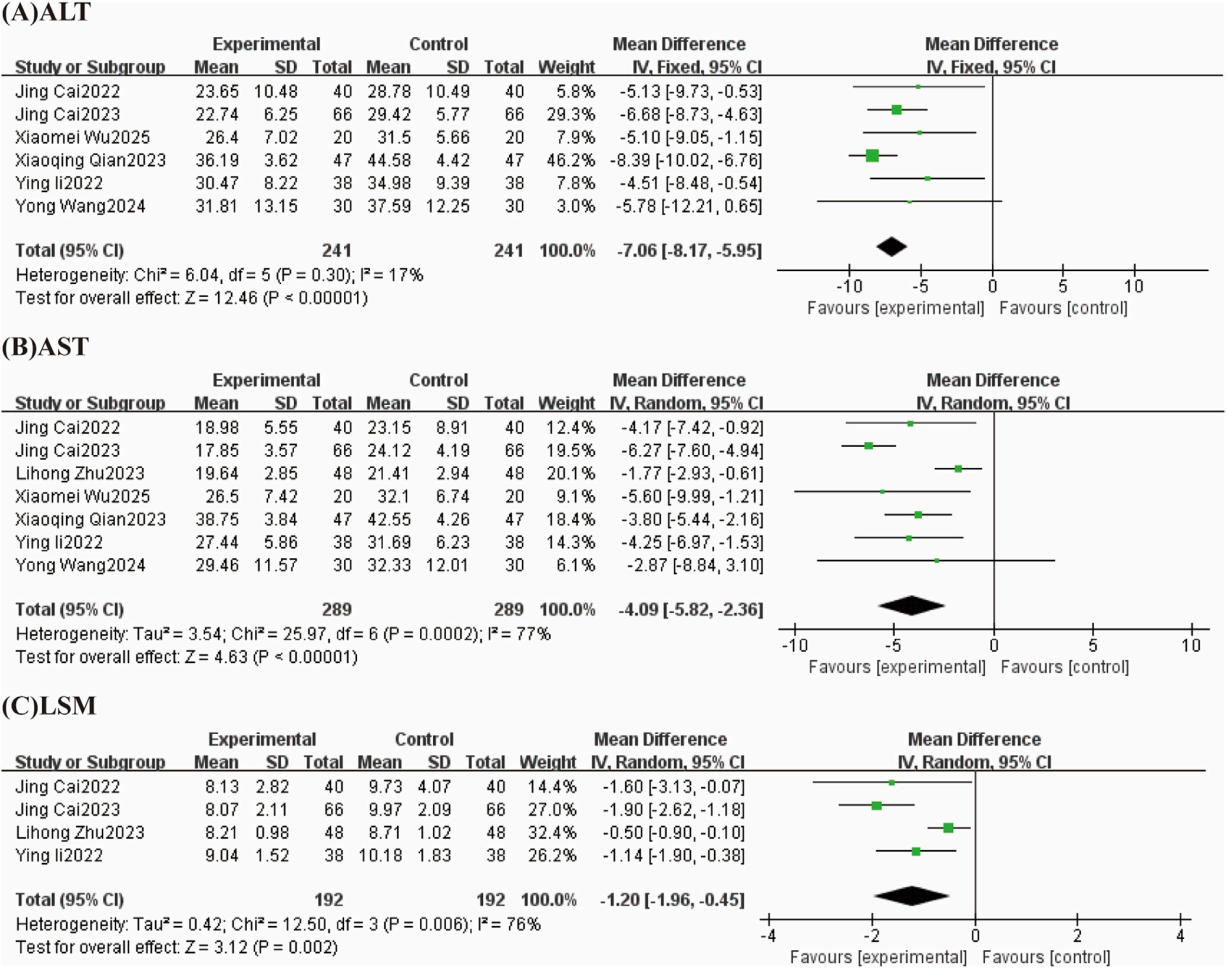
Forest plot (effect size and 95% confidence interval) summarizing the effect of TCM-preparation on three outcome measures: **(A)** ALT, **(B)** AST, **(C)** LSM.

Six studies reported ALT with low heterogeneity between studies (I^2^ = 17%, P = 0.30) using a fixed effect model. The results showed that TCM-preparation reduced ALT in patients with T2DM combined with MAFLD (MD = −7.06, CI [−8.17, −5.95], P < 0.00001) (Figure 7A).

##### 3.4.4.2 AST

Seven studies reported AST with a high degree of heterogeneity between studies (I^2^ = 77%, P = 0.0002), which were analyzed by Meta-analysis using a random-effects model. The overall study showed that TCM-preparation improved AST in patients with T2DM combined with MAFLD compared with controls (MD = −4.09, CI [−5.82, −2.36], P < 0.00001) (Figure 7B). To explore the source of heterogeneity, we performed subgroup analyses with intervention time (8 w, 12 w), and the results suggested that the treatment group interventions may not be the source of heterogeneity (Supplementary Figure S7). To assess the robustness of the results, we excluded articles one at a time and analyzed the remaining articles by sensitivity analysis, and found that heterogeneity was significantly reduced after the exclusion of Lihong Zhu 2023, and the combined effect sizes did not change significantly, suggesting that the results were stable (Table 3).

##### 3.4.4.3 LSM

Four studies reported LSM with large heterogeneity across studies (I^2^ = 76%, P = 0.006) using a random effects model. The results showed that TCM-preparation reduced LSM in patients with T2DM combined with MAFLD (MD = −1.20, CI [−1.96, −0.45], P = 0.002) (Figure 7C). To assess the robustness of the results, we excluded articles one at a time and analyzed the remaining articles by sensitivity analysis, and found that heterogeneity was significantly reduced after excluding Lihong Zhu 2023 and the effect size of the combination did not change significantly, suggesting that the results were stable (Table 3).

#### 3.4.5 Effect of TCM-preparation on BMI

**FIGURE 8:**
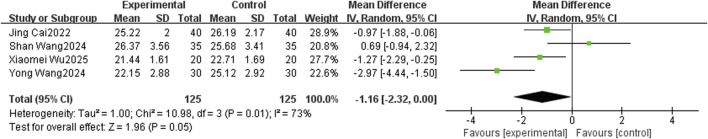
The forest plot (effect size and 95% confidence interval) summarizes the effect of TCM-preparation on BMI.

BMI was reported in 4 studies with large heterogeneity between studies (I^2^ = 73%, P = 0.01) using a random effects model. The results showed that TCM-preparation reduced BMI in patients with T2DM combined with MAFLD (MD = −1.16, C [−2.32, 0.00], P = 0.05) (Figure 8). To assess the robustness of the results, we excluded articles one by one and analyzed the remaining articles by sensitivity analysis, and found that the heterogeneity was significantly reduced after excluding Shan Wang 2024 and Yong Wang 2024, and the effect sizes for the combination were significantly changed, indicating that the results were stable (Table 3).

#### 3.4.6 Effective rate and adverse event rate

##### 3.4.6.1 Effective rate

**FIGURE 9:**
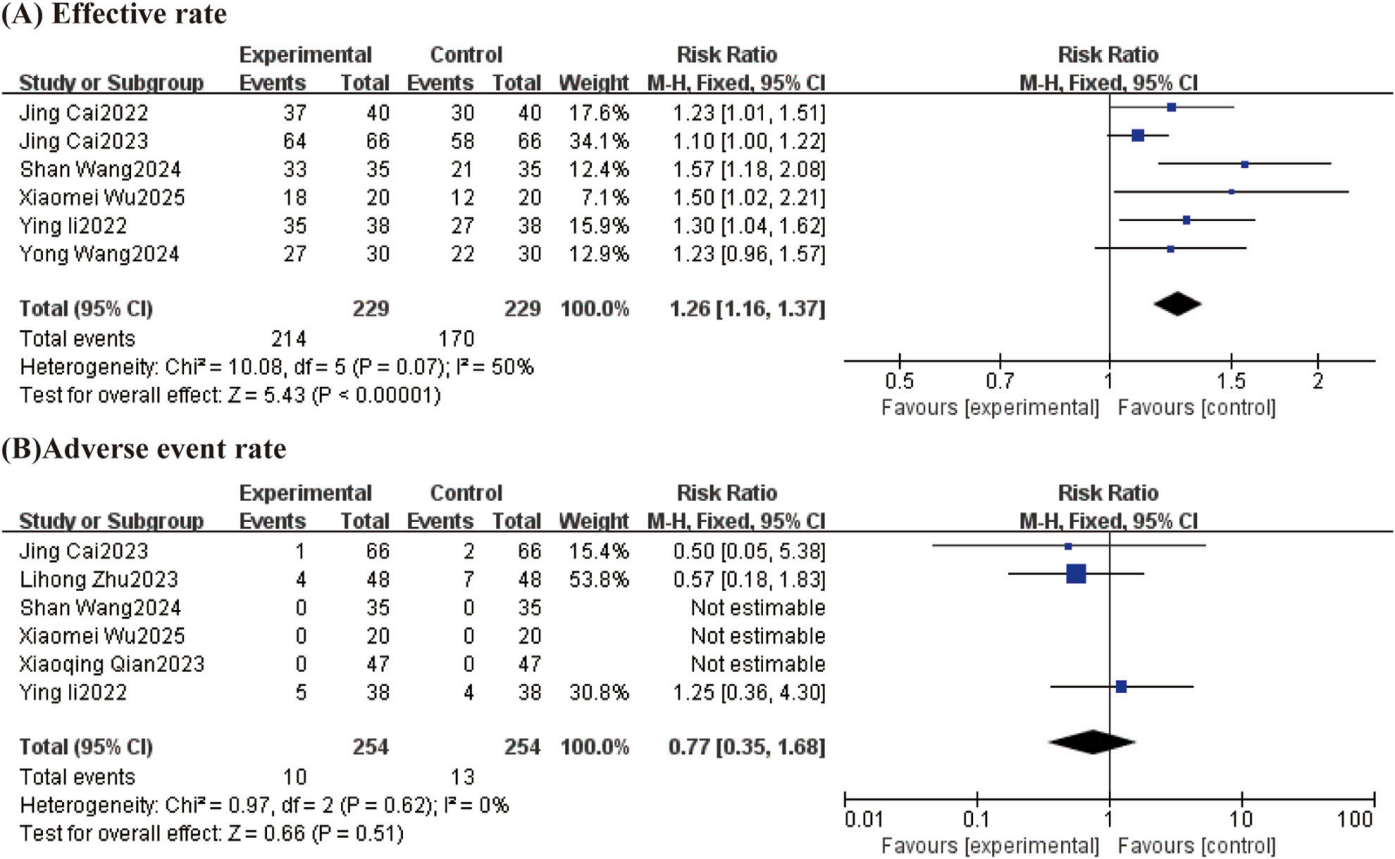
Forest plot (effect size and 95% confidence interval) summarizing the effect of TCM-preparation on two outcome measures: **(A)** Effective rate and **(B)** Adverse event rate.

Effective rates were reported in 6 studies, with general heterogeneity between studies (I^2^ = 50%, P = 0.07), using a fixed-effects model. The results showed that TCM-preparation reduced the effective rate in patients with T2DM combined with MAFLD (RR = 1.26, CI [1.16, 1.37], P = <0.00001) (Figure 9A).

##### 3.4.6.2 Adverse event rate

A total of six studies reported adverse reactions. One case of gastrointestinal reaction, two cases of hypoglycemia, and one case of malaise occurred in the Lihong Zhu 2023 study group; two cases of gastrointestinal reaction, one case of hypoglycemia, three cases of malaise, and one case of dizziness and headache occurred in the control group. No adverse reactions occurred in any of the Xiao-Qing Qian 2023 groups. Jing Cai 2023, the test group experienced 1 case of dizziness, and the control group experienced 1 case of dizziness and 1 case of nausea and vomiting. The remaining three studies showed no adverse events in either group. The results of the studies showed that the adverse event rate was comparable between the two groups (RR = 0.77, CI [0.35, 1.68]) without statistical significance (Figure 9B).

## 4 Discussion

### 4.1 Main results

Although some studies have confirmed that TCM-preparation can improve the glycolipid metabolism and liver function of T2DM combined with MAFLD (Peng et al., 2022), the present study focuses on TCM-preparation on steatosis in T2DM combined with MAFLD. The results of Meta-analysis showed that compared with TCM-preparation treatment, TCM-preparation treatment could effectively improve the CAP value, glucose-lipid metabolism level, liver enzymes, LSM and effective rate of patients with T2DM combined with MAFLD; the incidence of adverse events was comparable between the two groups.

Liver biopsy is used as a gold indicator for diagnosing liver steatosis and grading liver fibrosis, but it is an invasive test that causes mild pain and discomfort in many patients (Cadranel et al., 2000) and is expensive. Conventional ultrasound, as an alternative testing modality, has lower sensitivity and higher accuracy for steatosis >20% (Dasarathy et al., 2009). Fibroscan, a transient elastography device that measures CAP by acquiring ultrasound signals, has good sensitivity and specificity for steatosis, and can quantitatively measure the extent of steatosis and may be more accurate than liver biopsy (Machado and Cortez-Pinto, 2013). A study of 150 patients who underwent liver biopsy and CAP values on the same day supports the use of CAP scores as a non-invasive diagnostic modality for steatosis (Atzori et al., 2023). CAP may better reflect steatosis, and fat-induced IR may be an important mechanism for the high prevalence of MAFLD (Chon et al., 2016). Studies have also shown that high CAP values are associated with progression of liver fibrosis (Singh et al., 2015). Especially in patients with combined IR. Studies have confirmed that CAP values are also positively correlated with the amount of body fat in the limbs, trunk and viscera, with a stronger correlation with the amount of trunk and visceral fat (Chon et al., 2016), which suggests that CAP not only evaluates hepatic steatosis, but also reflects the whole-body fat distribution, and that higher CAP values also imply metabolic disorders and increased IR (Huang et al., 2021). The current study has shown that TCM-preparation can improve CAP value in patients with T2DM combined with MAFLD, reduce steatosis, and delay the progression of hepatic fibrosis, so lowering CAP value is beneficial for patients with MAFLD combined with T2DM.

The present study also found that TCM-preparation has very good efficacy in liver stiffness measurement (LSM), which is a noninvasive test that can reflect the degree of liver fibrosis and also correlate with the degree of steatosis, and the results of the present study also showed that TCM-preparation can also improve liver fibrosis.

Obesity is a major factor in the development of T2DM and MAFLD, which can drive the development of T2DM and MAFLD through IR (Zhao et al., 2023), lipotoxicity (Perez-Martinez et al., 2010), inflammation, and other mechanisms. The prevalence of T2DM and MAFLD is directly proportional to the increase in BMI (Younossi et al., 2019), and weight loss can lessen pancreatic fat accumulation and alleviate T2DM (Al-Mrabeh et al., 2020), and the current study also demonstrated that TCM-preparation could significantly reduce BMI in patients with T2DM combined with MAFLD. Meanwhile, in the existing study, it has been shown that in overweight/obese individuals, liver CAP values are more highly correlated with IR compared to other indicators of obesity outcomes (Li et al., 2024).

The efficacy of the Chinese medicines included in this study is consistent with the results of a previous data mining analysis (Dou et al., 2021), which mainly focuses on nourishing yin and tonifying the kidney, benefiting qi and strengthening the spleen, detoxifying the liver, and clearing heat and dampness. The most commonly used TCM compound in this study was Zinlida granules, which was confirmed in animal studies to have antioxidant effects, upregulate insulin signaling pathway, improve IR, reduce oxidative stress in the liver (Liu et al., 2015), antagonize hepatocyte apoptosis (Hao et al., 2022), and have good potential for the treatment of both T2DM and MAFLD. Ginseng-lingbaijusan can play an anti-inflammatory role by inhibiting the p38 MAPK pathway (Yang et al., 2014). It is consistent with the present study that TCM-preparation can improve glycolipid metabolism and steatosis in T2DM combined with MAFLD.

### 4.2 Strengths and limitations

The current study is a Meta-analysis to quantitatively assess the effect of TCM-preparation in the treatment of MADLD combined with T2DM, which provides better evidence support for researchers to understand this issue. The current study updated the latest research advances and included the CAP value as the main outcome indicator in addition to the common indicators of glucose-lipid metabolism and liver function, which allows for a multidimensional and multilevel assessment of the effectiveness of TCM-preparation in the treatment of MADLD combined with T2DM.

However, this study still has some limitations. First, the number of included studies was small, which may have led to biased results and prevented meta-regression analysis. Second, the methodological quality of the included studies was low, and some outcome measures showed high heterogeneity. Considering the unique characteristics of Chinese herbal medicine in terms of odor and texture, this may be related to the lack of use of a blinded method. Third, some studies did not report adverse reactions, so statistics could only be based on available data; however, the safety of Chinese herbal medicine still requires further confirmation. Fourth, the maximum intervention duration in the included studies was 12 weeks, and the long-term efficacy remains unclear. Fifth, this study only included TCM formulas and did not include single herbs or compounds, and their pharmacological effects require further exploration.

### 4.3 Implications for clinical practice and future research

Based on the findings of this study and the aforementioned limitations, we propose the following recommendations for future clinical research. First, we recommend conducting large-scale, multi-center clinical studies to enhance the reliability of clinical data. Second, clinical study protocols should be more detailed, with specific measures for randomization, blinding, and protocol confidentiality. Third, given the characteristics of the disease, prevention and delay of disease progression are the primary treatment objectives; therefore, we recommend extending the follow-up period to assess changes in the later stages of the disease. Fourth, clinical focus should be directed toward adverse reactions in patients, with pharmacology and toxicology experiments conducted to identify their causes and explore appropriate combinations and dosages to mitigate their occurrence. Fifth, through experimental research, the primary active components and compounds of the aforementioned TCM-preparation should be identified, and their specific mechanisms of action in treating the disease should be explored.

## 5 Conclusion

The present study shows that TCM-preparation can reduce the deposition of liver fat in T2DM combined with MAFLD, restore the function of hepatocytes, and have a good therapeutic effect on the level of glucose and lipid metabolism and liver function. However, the present study has some limitations, and some high-quality, multicenter, large-sample clinical studies are urgently needed next to make up for the shortcomings of the present study.

## Data Availability

The original contributions presented in the study are included in the article/Supplementary Material, further inquiries can be directed to the corresponding authors.

## References

[B1] Aguirre F. Brown A. Cho N. H. Dahlquist G. Dodd S. Dunning T. (2013). IDF diabetes atlas. 6th ed. Brussels: International Diabetes Federation. 10.1016/S0140-6736(13)62229-1

[B2] Al-Mrabeh A. Hollingsworth K. G. Shaw J. A. McConnachie A. Sattar N. Lean M. E. (2020). 2-year remission of type 2 diabetes and pancreas morphology: a post-hoc analysis of the DiRECT open-label, cluster-randomised trial. Lancet Diabetes Endocrinol. 8 (12), 939–948. 10.1016/s2213-8587(20)30303-x 33031736

[B3] Atzori S. Pasha Y. Maurice J. B. Taylor-Robinson S. D. Campbell L. Lim A. K. P. (2023). The accuracy of ultrasound controlled attenuation parameter in diagnosing hepatic fat content. Hepat. Med. 15, 51–61. 10.2147/HMER.S411619 37325088 PMC10263157

[B4] Cadranel J. F. Rufat P. Degos F. (2000). Practices of liver biopsy in France: results of a prospective nationwide survey. For the group of epidemiology of the French association for the study of the liver (AFEF). Group Epidemiol. Fr. Assoc. Study Liver (AFEF). Hepatology32 32 (3), 477–481. 10.1053/jhep.2000.16602 10960438

[B5] Cai J. Z. Zheng Z. K. Zhang M. Y. Xia X. Wei M. (2022). Clinical efficacy of Jinlida granules combined with liraglutide in the treatment of type 2 diabetes mellitus complicated with metabolic-related fatty liver disease. Chin. J. Difficult Complicat. Cases 21, 399–403. 10.3969/j.issn.1671-6450.2022.04.013

[B6] Cai J. Xia X. Qiao J. Gao Y. Li H. Liu Y. (2023). Effect of jinlida granule on glycolipid metabolism, oxidative stress, and inflammatory factors in type 2 diabetes mellitus patients with non-alcoholic fatty liver disease. Trop. J. Pharm. Res. 22, 597–603. 10.4314/tjpr.v22i3.17

[B7] Castéra L. Hézode C. Roudot-Thoraval F. Bastie A. Zafrani E. S. Pawlotsky J. M. (2003). Worsening of steatosis is an independent factor of fibrosis progression in untreated patients with chronic hepatitis C and paired liver biopsies. Gut52 52 (2), 288–292. 10.1136/gut.52.2.288 PMC1774979 12524415

[B8] Chon Y. E. Kim K. J. Jung K. S. Kim S. U. Park J. Y. Kim D. Y. (2016). The relationship between type 2 diabetes mellitus and non-alcoholic fatty liver disease measured by controlled attenuation parameter. Yonsei Med. J. 57 (4), 885–892. 10.3349/ymj.2016.57.4.885 27189281 PMC4951464

[B9] Dai X. Feng J. Chen Y. Huang S. Shi X. Liu X. (2021). Traditional Chinese medicine in nonalcoholic fatty liver disease: molecular insights and therapeutic perspectives. Chin. Med. 16, 68. 10.1186/s13020-021-00469-4 34344394 PMC8330116

[B10] Dasarathy S. Dasarathy J. Khiyami A. Joseph R. Lopez R. McCullough A. J. (2009). Validity of real-time ultrasound in the diagnosis of hepatic steatosis: a prospective study. J. Hepatol. 51 (6), 1061–1067. 10.1016/j.jhep.2009.09.001 19846234 PMC6136148

[B11] de Lédinghen V. Vergniol J. Capdepont M. Chermak F. Hiriart J. B. Cassinotto C. (2014). Controlled attenuation parameter (CAP) for the diagnosis of steatosis: a prospective study of 5323 examinations. J. Hepatol. 60 (5), 1026–1031. 10.1016/j.jhep.2013.12.018 24378529

[B12] Dou Z. Xia Y. Zhang J. Li Y. Zhang Y. Zhao L. (2021). Syndrome differentiation and treatment regularity in traditional Chinese medicine for type 2 diabetes: a text mining analysis. Front. Endocrinol. 12, 728032. 10.3389/fendo.2021.728032 PMC8733618 35002950

[B13] Eslam M. Newsome P. N. Sarin S. K. Anstee Q. M. Targher G. Romero-Gomez M. (2020). A new definition for metabolic dysfunction-associated fatty liver disease: an international expert consensus statement. J. Hepatol. 73 (1), 202–209. 10.1016/j.jhep.2020.03.039 32278004

[B14] Hao Y. Y. Cui W. W. Gao H. L. Wang M. Y. Liu Y. Li C. R. (2022). Jinlida granules ameliorate the high-fat-diet induced liver injury in mice by antagonising hepatocytes pyroptosis. Pharm. Biol. 60 (1), 274–281. 10.1080/13880209.2022.2029501 35138995 PMC8843117

[B15] Huang Z. Ng K. Chen H. Deng W. Li Y. (2021). Validation of controlled attenuation parameter measured by FibroScan as a novel surrogate marker for the evaluation of metabolic derangement. Front. Endocrinol. 12, 739875. 10.3389/fendo.2021.739875 PMC8841525 35173677

[B16] Karlas T. Petroff D. Sasso M. Fan J. G. Mi Y. Q. de Lédinghen V. (2017). Individual patient data meta-analysis of controlled attenuation parameter (CAP) technology for assessing steatosis. J. Hepatol. 66 (5), 1022–1030. 10.1016/j.jhep.2016.12.022 28039099

[B17] Kotronen A. Juurinen L. Hakkarainen A. Westerbacka J. Cornér A. Bergholm R. (2008). Liver fat is increased in type 2 diabetic patients and underestimated by serum alanine aminotransferase compared with equally Obese nondiabetic subjects. Diabetes Care 31 (1), 165–169. 10.2337/dc07-1463 17934148

[B18] Leandro G. Mangia A. Hui J. Fabris P. Rubbia-Brandt L. Colloredo G. (2006). Relationship between steatosis, inflammation, and fibrosis in chronic hepatitis C: a meta-analysis of individual patient data. Gastroenterology 130 (6), 1636–1642. 10.1053/j.gastro.2006.03.014 16697727

[B19] Leite N. C. Villela-Nogueira C. A. Cardoso C. R. Salles G. F. (2014). Non-alcoholic fatty liver disease and diabetes: from physiopathological interplay to diagnosis and treatment. World J. Gastroenterol. 20 (26), 8377–8392. 10.3748/wjg.v20.i26.8377 25024596 PMC4093691

[B20] Li X. Geng-Ji J. J. Quan Y. Y. Qi L. M. Sun Q. Huang Q. (2022). Role of potential bioactive metabolites from traditional Chinese medicine for type 2 diabetes mellitus: an overview. Front. Pharmacol. 13, 1023713. 10.3389/fphar.2022.1023713 36479195 PMC9719995

[B21] Li Y. Cao W. F. Li J. D. (2022). Effect of shenmai lanling decoction on liver fat content and fibrosis in type 2 diabetes mellitus with non-alcoholic fatty liver disease. Chin. J. Tradit. Chin. Med. 40, 144–148. 10.13193/j.issn.1673-7717.2022.06.032

[B22] Li Z. Liu R. Gao X. Hou D. Leng M. Zhang Y. (2024). The correlation between hepatic controlled attenuation parameter (CAP) value and insulin resistance (IR) was stronger than that between body mass index, visceral fat area and IR. Diabetol. Metab. Syndr. 16 (1), 153. 10.1186/s13098-024-01399-5 38982535 PMC11232147

[B23] Liu Y. Song A. Zang S. Wang C. Song G. Li X. (2015). Jinlida reduces insulin resistance and ameliorates liver oxidative stress in high-fat fed rats. J. Ethnopharmacol. 162, 244–252. 10.1016/j.jep.2014.12.040 25577992

[B24] Machado M. V. Cortez-Pinto H. (2013). Non-invasive diagnosis of non-alcoholic fatty liver disease. A critical appraisal. J. Hepatol. 58 (5), 1007–1019. 10.1016/j.jhep.2012.11.021 23183525

[B25] Ni Y. Wu X. Yao W. Zhang Y. Chen J. Ding X. (2024). Evidence of traditional Chinese medicine for treating type 2 diabetes mellitus: from molecular mechanisms to clinical efficacy. Pharm. Biol. 62 (1), 592–606. 10.1080/13880209.2024.2374794 39028269 PMC11262228

[B26] Peng S. Liu L. Xie Z. Zhang X. Xie C. Ye S. (2022). Chinese herbal medicine for type 2 diabetes mellitus with nonalcoholic fatty liver disease: a systematic review and meta-analysis. Front. Pharmacol. 13, 863839. 10.3389/fphar.2022.863839 35833030 PMC9271569

[B27] Perez-Martinez P. Perez-Jimenez F. Lopez-Miranda J. (2010). n-3 PUFA and lipotoxicity. Biochim. Biophys. Acta 1801 (3), 362–366. 10.1016/j.bbalip.2009.09.010 19781663

[B28] Qian X. Q. Zhou J. W. Li S. G. Yuan S. F. (2023). Effect of Huanglian Wendan decoction on blood glucose and lipid levels in patients with type 2 diabetes mellitus and non-alcoholic fatty liver disease of phlegm-heat syndrome. Liaoning J. Tradit. Chin. Med. 50, 155–159. 10.13192/j.issn.1000-1719.2023.09.042

[B29] Singh S. Allen A. M. Wang Z. Prokop L. J. Murad M. H. Loomba R. (2015). Fibrosis progression in nonalcoholic fatty liver vs nonalcoholic steatohepatitis: a systematic review and meta-analysis of paired-biopsy studies. Clin. Gastroenterol. Hepatol. 13 (4), 643–e40. 10.1016/j.cgh.2014.04.014 24768810 PMC4208976

[B30] Stefan N. Cusi K. (2022). A global view of the interplay between non-alcoholic fatty liver disease and diabetes. Lancet Diabetes Endocrinol. 10 (4), 284–296. 10.1016/S2213-8587(22)00003-1 35183303

[B31] Thiele M. Rausch V. Fluhr G. Kjærgaard M. Piecha F. Mueller J. (2018). Controlled attenuation parameter and alcoholic hepatic steatosis: diagnostic accuracy and role of alcohol detoxification. J. Hepatol. 68 (5), 1025–1032. 10.1016/j.jhep.2017.12.029 29343427

[B32] Tian H. Deng X. Ling Y. (2023). Predictive value of hepatic steatosis related indexes in type 2 diabetespatients with non-alcoholic fatty liver disease. J. Jiangsu University 33, 521–527. 10.13312/j.issn.1671-7783.y230120

[B33] Wang Y. Dai Z. Wang Q. He Y. Peng Y. Wu M. (2022). Clinical application of traditional Chinese medicine therapy for type 2 diabetes mellitus: an evidence map. Evid. Based Complement. Altern. Med. 2022 (1), 2755332. 10.1155/2022/2755332 PMC9325645 35911140

[B34] Wang Y. Zhang N. Wang L. W. Qi Y. Y. Liu J. Y. Zhang X. K. (2024). Clinical observation of wuling powder in treating type 2 diabetes mellitus with metabolic-associated fatty liver disease. Shanxi J. Tradit. Chin. Med. 40, 15–18. 10.20002/j.issn.1000-7156.2024.02.007

[B35] Wang S. Tang R. M. Huang J. K. Yang X. H. (2024). Clinical efficacy of yixiao formula in treating type 2 diabetes mellitus with non-alcoholic fatty liver disease of Qi-Yin deficiency syndrome. China J. Tradit. Chin. Med. 39, 6250–6255. 10.13193/j.issn.1673-7717.2024.10.012

[B36] Wong V. W. Ekstedt M. Wong G. L. Hagström H. (2023). Changing epidemiology, global trends and implications for outcomes of NAFLD. J. Hepatol. 79 (3), 842–852. 10.1016/j.jhep.2023.04.036 37169151

[B37] Wu W. Xia Q. Guo Y. Wang H. Dong H. Lu F. (2023). Berberine enhances the function of db/db mice islet β cell through GLP-1/GLP-1R/PKA signaling pathway in intestinal L cell and islet α cell. Front. Pharmacol. 14, 1228722. 10.3389/fphar.2023.1228722 37469873 PMC10352779

[B38] Wu X. M. Li K. Y. Wang G. Q. Zhao Y. M. Zhao Q. Huang J. (2025). Clinical study of Quzhi Jiangtang formula in treating non-alcoholic fatty liver disease with type 2 diabetes mellitus of liver stagnation-spleen deficiency and damp-heat syndrome. J. Info. Tradit. Chin. Med. 42, 56–61. 10.19656/j.cnki.1002-2406.20250109

[B39] Xu J. Piao C. Qu Y. Liu T. Peng Y. Li Q. (2022). Efficacy and mechanism of Jiedu Tongluo Tiaogan Formula in treating type 2 diabetes mellitus combined with non-alcoholic fatty liver disease: study protocol for a parallel-armed, randomized controlled trial. Front. Pharmacol. 13, 924021. 10.3389/fphar.2022.924021 36034810 PMC9411737

[B40] Yang Q. H. Xu Y. J. Liu Y. Z. Liang Y. J. Feng G. F. Zhang Y. P. (2014). Effects of Chaihu-Shugan-San and shen-ling-bai-zhu-san on p38 MAPK pathway in kupffer cells of nonalcoholic steatohepatitis. Evid. Based Complement. Altern. Med. 2014 (1), 671013. 10.1155/2014/671013 PMC3984846 24795769

[B41] Yanni Z. Chang F. Lianguo L. Zongcun C. (2024). Research progress on the role of abnormal visceral fat accumulation in the occurrence and development of diabetic complications. Shandong Med. J. 64 (2), 105–108. 10.3969/j.issn.1002-266X.2024.02.024

[B42] Yin J. Huang Y. Wang K. Zhong Q. Liu Y. Ji Z. (2024). Ginseng extract improves pancreatic islet injury and promotes β-cell regeneration in T2DM mice. Front. Pharmacol. 15, 1407200. 10.3389/fphar.2024.1407200 38989151 PMC11234855

[B43] Younossi Z. Anstee Q. M. Marietti M. Hardy T. Henry L. Eslam M. (2018). Global burden of NAFLD and NASH: trends, predictions, risk factors and prevention. Nat. Rev. Gastroenterol. Hepatol. 15 (1), 11–20. 10.1038/nrgastro.2017.109 28930295

[B44] Younossi Z. Tacke F. Arrese M. Chander Sharma B. Mostafa I. Bugianesi E. (2019). Global perspectives on nonalcoholic fatty liver disease and nonalcoholic steatohepatitis. Hepatology 69 (6), 2672–2682. 10.1002/hep.30251 30179269

[B45] Zhao X. An X. Yang C. Sun W. Ji H. Lian F. (2023). The crucial role and mechanism of insulin resistance in metabolic disease. Front. Endocrinol. 14, 1149239. 10.3389/fendo.2023.1149239 PMC10086443 37056675

[B46] Zhu L. H. (2023). Clinical study of Shenling Baizhu powder combined with Erchen decoction and exercise therapy in treating diabetes with non-alcoholic fatty liver. Sichuan J. Tradit. Chin. Med. 41, 132–135. 10.3969/j.issn.1000-3649.2023.07.031

