# Supplementary figures and images for: Metabolic dysfunction-associated fatty liver in type 2 diabetes mellitus patient: can a systematic review of and meta-analysis of commonly used TCM-preparation shed light on their efficacy?
Source: Front Pharmacol. 2025 Aug 14;16:1578371. doi: 10.3389/fphar.2025.1578371 (PMC12390981; doi:10.3389/fphar.2025.1578371)

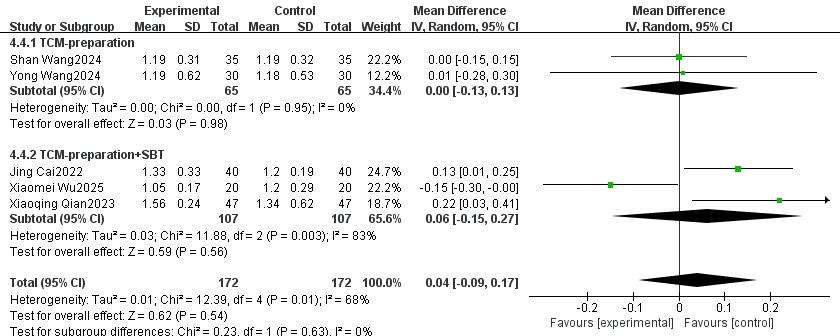

Supplement: Supplementary file 2 [file Image6.tif]

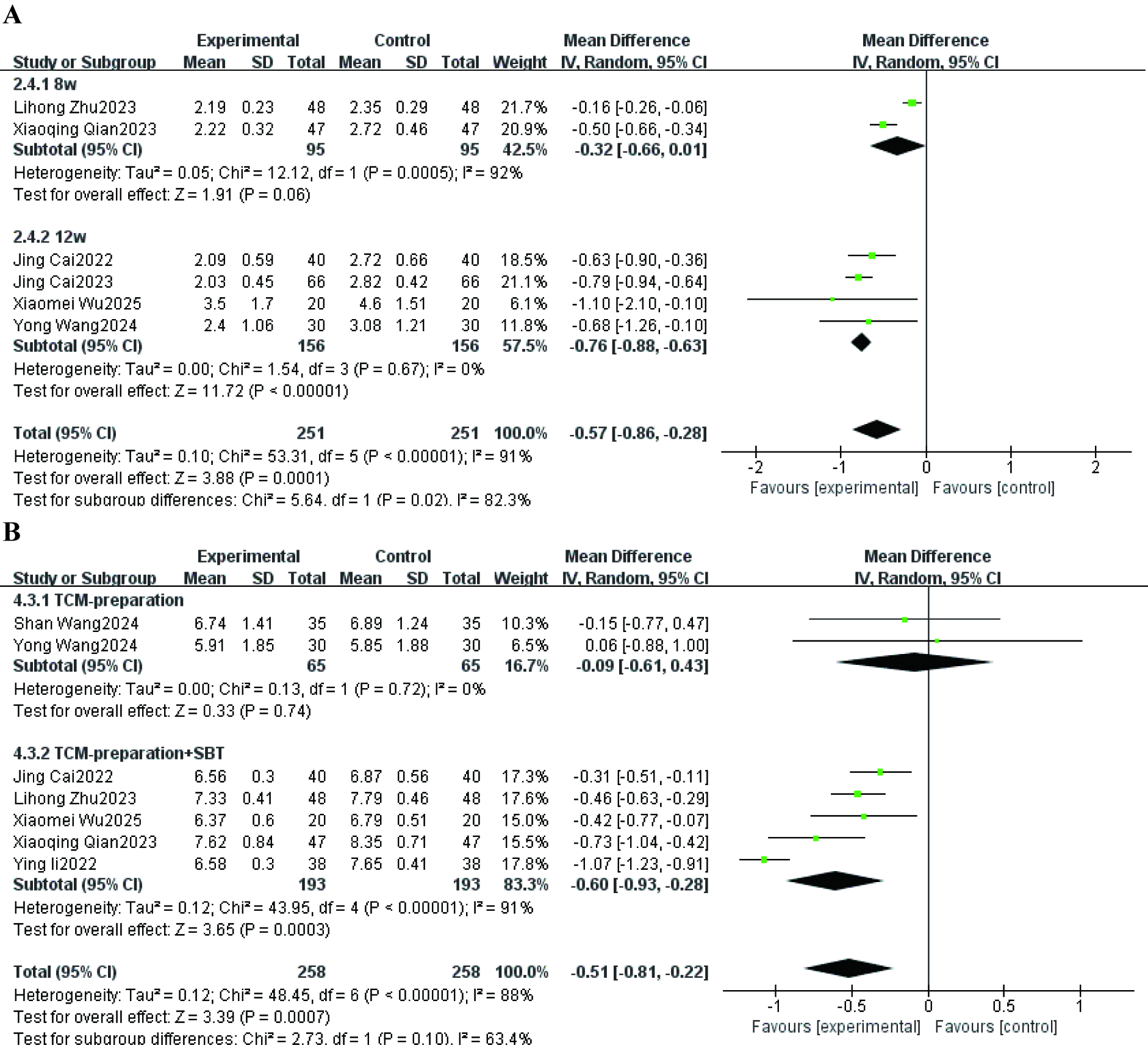

Supplement: Supplementary file 3 [file Image3.tif]

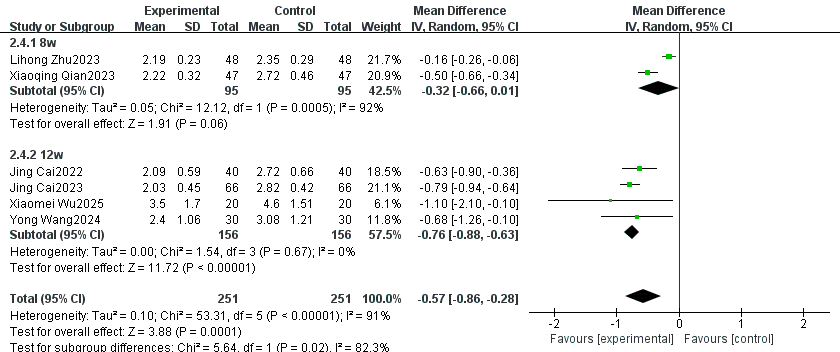

Supplement: Supplementary file 4 [file Image4.tif]

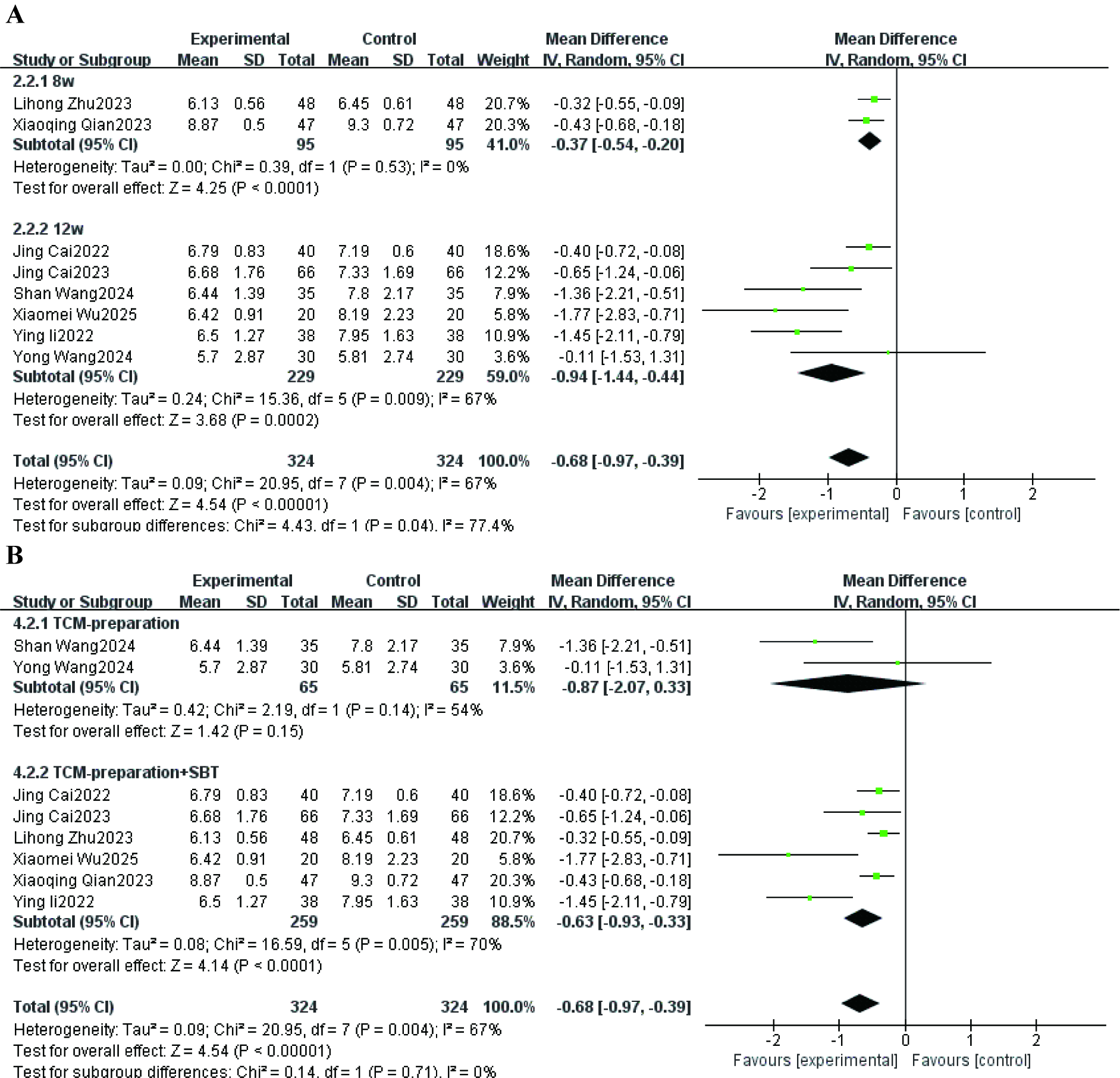

Supplement: Supplementary file 5 [file Image2.tif]

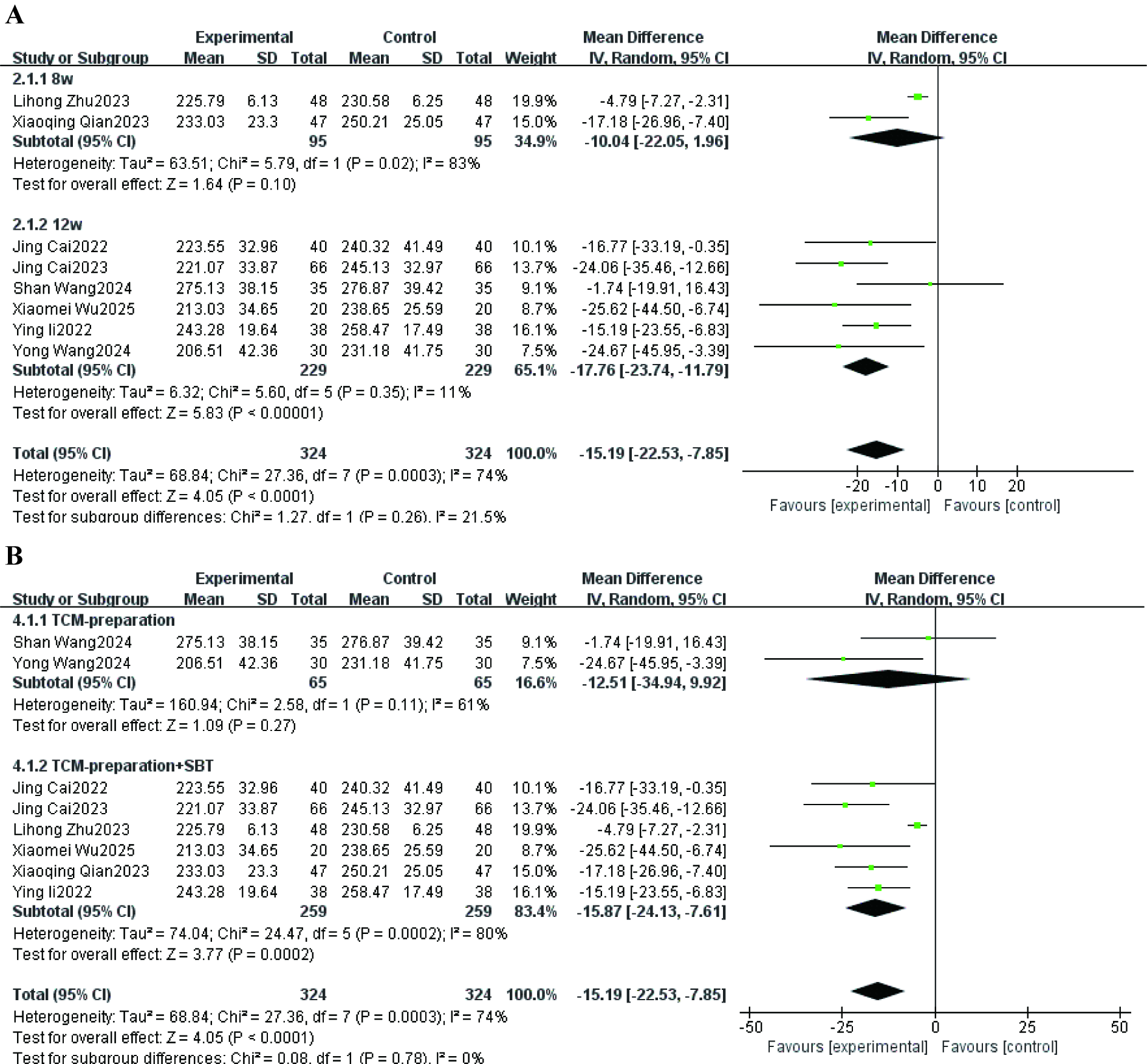

Supplement: Supplementary file 6 [file Image1.tif]

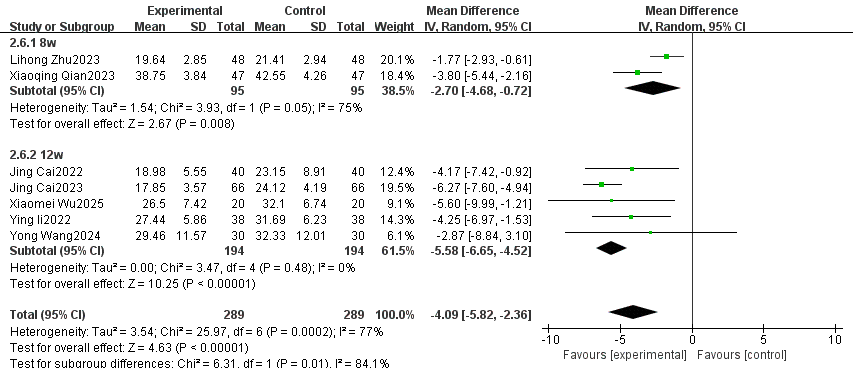

Supplement: Supplementary file 7 [file Image7.tif]

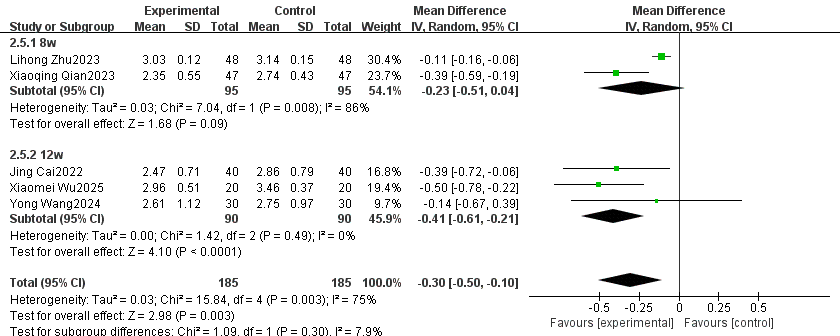

Supplement: Supplementary file 9 [file Image5.tif]
